# Prevalence and proliferation of antibiotic resistance genes in the subtropical mangrove wetland ecosystem of South China Sea

**DOI:** 10.1002/mbo3.871

**Published:** 2019-06-28

**Authors:** Huaxian Zhao, Bing Yan, Xueyan Mo, Pu Li, Baoqin Li, Quanwen Li, Nan Li, Shuming Mo, Qian Ou, Peihong Shen, Bo Wu, Chengjian Jiang

**Affiliations:** ^1^ Guangxi Key Lab of Mangrove Conservation and Utilization Guangxi Mangrove Research Center Guangxi Academy of Sciences Beihai China; ^2^ Guangxi Key Laboratory of Marine Natural Products and Combinatorial Biosynthesis Chemistry Guangxi Academy of Sciences Nanning China; ^3^ State Key Laboratory for Conservation and Utilization of Subtropical Agro‐Bioresources, College of Life Science and Technology Guangxi University Nanning China; ^4^ PFOMIC Bioinformatics Company Nanning China; ^5^ Guangdong Key Laboratory of Integrated Agro‐Environmental Pollution Control and Management Guangdong Institute of Eco‐Environmental Science & Technology Guangzhou China; ^6^ Key Laboratory of Environment Change and Resources Use in Beibu Gulf, Ministry of Education (Nanning Normal University) Nanning China; ^7^ Department of chemical and biological engineering Guangxi Normal University for Nationalities Chongzuo China

**Keywords:** antibiotic resistance genes, mangrove wetland, microbial community, mobile genetic elements, sediment properties

## Abstract

The emerging pollutants antibiotic resistance genes (ARGs) are prevalent in aquatic environments such as estuary. Coastal mangrove ecosystems always serve as natural wetlands for receiving sewage which always carry ARGs. Currently, the research considering ARG distribution in mangrove ecosystems gains more interest. In this work, we investigated the diversity of ARGs in an urban estuary containing mangrove and nonmangrove areas of the South China Sea. A total of 163 ARGs that classified into 22 resistance types and six resistance mechanisms were found. ARG abundance of the samples in the estuary is between 0.144 and 0.203. This is within the general range of Chinese estuaries. The difference analysis showed that abundances of total ARGs, six most abundant ARGs (*mtrA*, *rpoB*, *rpoC*, *rpsL*, *ef‐Tu*, and *parY*), the most abundant resistance types (elfamycin, multidrug, and peptide), and the most abundant resistance mechanism (target alteration) were significantly lower in mangrove sediment than that in nonmangrove sediment (*p* < 0.05). Network and partial redundancy analysis showed that sediment properties and mobile genetic elements were the most influential factors impacting ARG distribution rather than microbial community. The two factors collectively explain 51.22% of the differences of ARG distribution. Our study indicated that mangrove sediments have the capacity to remove ARGs. This work provides a research paradigm for analysis of ARG prevalence and proliferation in the subtropical marine coastal mangrove ecosystem.

## Graphical abstract

We investigated the diversity and functions of antibiotic resistance genes (ARGs) in an urban estuary containing mangroves in the South China Sea by shotgun metagenomics method. Total abundance of ARGs, six ARGs mtrA, rpoB, rpoC, rpsL, ef‐Tu, and parY, abundance of resistance types elfamycin, multidrug and peptide, and resistance mechanism target alteration were all significantly lower in mangrove sediment samples than that in nonmangrove sediment. Network and partial redundancy analysis showed that sediment properties and mobile genetic elements (MGEs) were the most influential factors impacting ARG distributio. Our study indicated that mangrove sediments have the capacity to remove ARGs.

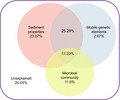

## 1 INTRODUCTION

Antibiotics have effectively helped humans fight bacterial infections for many years, but the increase in antibiotic resistance occurrence in clinically relevant microorganisms has rendered ineffective antibiotics. Around the world, antibiotic resistance is rising to a dangerously high level, leading to increased medical costs, bacterial infections, and mortality. The problem of antibiotic abuse and antibiotic resistance in China is quite serious. Antibiotic use per capita in China is more than five times than that of Europe and the United States (Zhang, Ying, Pan, Liu, & Zhao, 2015). In China alone, roughly 24,748 tons of antibiotics from human and animal sources were released in natural waters in 2013 (Zhang et al., 2015). This exacerbates the problem of antibiotic resistance in aquatic ecosystems.

Extensive researches show that different levels of antibiotic resistance genes (ARGs) exist in various aquatic environments, such as sewage treatment plants (Munir, Wong, & Xagoraraki, 2011), rivers (Ouyang, Huang, Zhao, Li, & Su, 2015), plateau lakes (Chen, Yuan, et al., 2016), constructed wetlands (Fang et al., 2017), and coastal industrial mariculture systems (Wang et al., 2018). Investigation of ARGs in 18 estuarine sediments across 4,000 km of coastal waters in China shows that there are 1 × 10^6^–1 × 10^8^ copies of ARGs per 1 g of sediment (Zhu et al., 2017). Because ARGs may spread in microorganisms through horizontal gene transfer (HGT) (Summers, 2006), the transmission pattern of ARGs in microbe‐rich aquatic environments, such as sewage treatment plants (Di Cesare et al., 2016; Mao et al., 2015; Munir et al., 2011) and constructed wetlands (Berglund et al., 2014; Nõlvak et al., 2013) is receiving considerable attention. However, few research focus on the fate of ARGs in mangrove, where is a common, microbe‐rich natural wetland.

Mangrove ecosystems are widely distributed in the intertidal zones of tropical and subtropical coasts, and the largest amount (42%) of the world's mangroves is in Asia (Giri et al., 2011). These ecosystems are especially essential for the global carbon cycle, and have important economic value to human society (Bouillon et al., 2008; Brander et al., 2012; Giri et al., 2011). They are composed of highly coordinated mangrove plants, animals, and microorganisms (Andreote et al., 2012; Bai et al., 2013; Holguin, Vazquez, & Bashan, 2001). Microorganisms in mangrove sediments are enriched during the process of mangrove succession (Chen, Zhao, Li, Jian, & Ren, 2016), and play important functions, such as carbon fixation and degradation, nitrogen fixation, sulfur metabolism, and sediment stabilization (Andreote et al., 2012; Bai et al., 2013; Holguin et al., 2001). Mangrove also receives sewage or aquaculture wastewater in coastal regions. The pollutants in water are removed mainly by tidal scour, plant assimilation, or microbial metabolism (Ouyang & Guo, 2016). Thus, mangroves are often considered as an economical alternative to sewage treatment plants because of their potential in removing and tolerating pollution. However, previous studies have not taken into account their potential in removing ARGs (Leung, Cai, & Tam, 2016; Ouyang & Guo, 2016; Wong, Tam, & Lan, 1997; Wu, Tam, & Wong, 2008). To our knowledge, some literatures reported that novel compounds as potential new antibiotics (Azman, Othman, Velu, Chan, & Lee, 2015) produce from *Actinobacteria* and antibiotic resistance microbes (Cabral et al., 2016; Ghaderpour et al., 2015; Jalal et al., 2010; Le, Munekage, & Kato, 2005) in the mangrove sediments, but few research study on distribution pattern of ARGs in the mangrove ecosystem.

Therefore, in this work, we analyzed the abundance and diversity of ARGs in the mangrove ecosystem located in an urban estuary of a city in the South China Sea. Via shotgun metagenomics approach, one of the appropriate methods to study the ARG diversity of environmental samples such as sediment, soil, water, etc. (Chen et al., 2013; Chen, Yuan, et al., 2016; Zinicola et al., 2015), we investigated the following contents: (a) antibiotic resistance level of the subtropical city estuary, (b) profiles of ARGs in mangrove and nonmangrove areas that both receive the same municipal wastewater, (c) correlations between ARGs and abiotic and biological factors in the sediments, (d) distribution pattern of antibiotic resistance, and (e) impacts of mangrove on the distribution of ARGs. Our work provides a research paradigm and theoretical support for analysis of ARG prevalence and proliferation in the subtropical marine coastal mangrove ecosystem.

## 2 MATERIALS AND METHODS

### 2.1 Sampling sites

**Figure 1:**
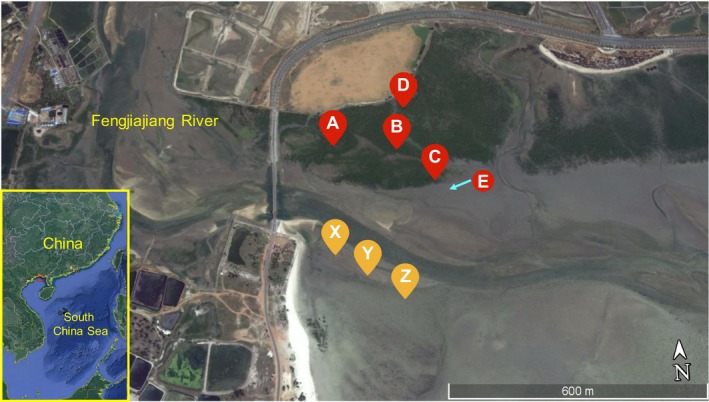
Geographic map of sampling sites. The red labels are the sampling sites that located at the north bank of the estuary, the A, B, C, and D are mangrove sampling sites, and the E is a sandy beach site outside the mangrove. The yellow labels are the south bank sites, all of them are sandy beach sites

The sampling location was the estuary of Fengjiajiang River in Beihai City, Guangxi Province, China (21°24′43.43″N, 109°9′50.98″E), which is surrounded by mangrove and a beach on two sides (Figure 1). The Fengjiajiang River receives municipal sewage, illegally discharged aquaculture sewage, and domestic sewage. The south bank is a beach with no plants, and the sediment type is sandy soil. The northern bank is covered by mangroves, most of which are identified as *Avicennia marina*; its sediment type is sandy or sandy loam. Outside the mangrove is a sandy beach. Samples were collected in the mangrove area from inner sites A and D, center site B, and outer site C. Samples were also collected from site E outside the mangrove area. Samples from the nonmangrove area were X, Y, and Z, which were located from the inner to the outer site. Figure 1 shows the geographic map of the sampling sites.

### 2.2 Sampling collection

All the sediment samples were collected on August 7, 2017. Sterile polyvinyl chloride tubes with a diameter of 3 cm and a height of 15 cm were used to collect samples from the sampling sites, each with a quadrat of 20 × 20 m. Five sediment samples were collected from each site and placed in a sterile bag inside a box filled with ice immediately after sufficient mixing. All samples were stored at −80°C in the same day.

### 2.3 DNA extraction and high‐throughput sequencing

DNA was extracted from sediments using the FastDNA SPIN Kit for Soil (MP Biomedicals) according to the manufacturer's protocols. The quality of the extracted DNA was verified by electrophoresis on 0.85% agarose gel and further quantified with a NanoDrop 2000 Spectrophotometer (Thermo Scientific). At least 6 μg of DNA from each sample was submitted to the Shanghai Majorbio Bio‐Pharm Technology Co., Ltd. (Shanghai, China) for sequencing. The DNA pools were prepared for sequencing and the size and quantity of the DNA library were assessed on Agilent 2,100 Bioanalyzer (Agilent) and with the Library Quantification Kit for Illumina (Kapa Biosciences, Woburn, MA), respectively. The library preparations were sequenced on an Illumina HiSeq 4,000 platform and paired‐end reads were generated.

The data output from each DNA sample was more than 5 Gb. The metagenomic sequencing data were deposited in the NCBI SRA database under the BioProject PRJNA472209.

### 2.4 Sediment property analysis

Sediment properties, such as pH, total organic carbon (TOC), and metal elements (Cu, Zn, Hg, Cd, and metalloid As), were determined using Chinese standard methods (Ministry of Agriculture of the People's Republic of China, 2006, 2007; Ministry of Ecology & Environment of the People's Republic of China, 1997, 2016; Standardization Administration of the People’s Republic of China, 2008).

### 2.5 Bioinformatic analysis

Based on previous studies, adapter sequences were removed using SeqPrep v1.33. Moreover, sequences <100 bp and those with quality <20 as well as reads containing an N base were removed using Sickle v1.2; then, clean reads were created (Yang, Sun, Yang, & Li, 2014). Nonbacteria sequences (algae, protist, etc) were also cleaned by local Blastn program against NR database (V20151204) from NCBI with evalue 1e‐10 (Camacho et al., 2009).

Function blastx (set as ‐‐evalue 1e‐5 –query‐cover 75 ‐‐id 90 ‐k 1 ‐‐compress 1) in DIAMOND v0.9.14 (Buchfink, Xie, & Huson, 2015) was executed to align the clean reads against the Comprehensive Antibiotic Resistance Database (CARD) (Jia et al., 2017) to annotate the ARG reads. ARGs were classified according to Antibiotic Resistance Ontology terms “antibiotic molecule” and “mechanism of antibiotic resistance” of CARD, which are called “resistance type” and “resistance mechanism” in this study, respectively. The multiple resistance genes were classified into three resistance types: (a) sulfonamide and sulfone; (b) macrolide‐lincosamide‐streptogramin; and (c) multidrug, other multiple resistance genes. The mobile genetic elements (MGEs) integrase and insertion sequence common region (ISCR) were annotated using the same method as that of ARGs, and the annotation of plasmids was according to a previous study (Chen et al., 2013).

16S rRNA reads from the Silva rRNA database were annotated by SortMeRna v2.1b (Kopylova, Noé, & Touzet, 2012; Quast et al., 2012).

The abundance of ARGs or MGEs was indicated as the ratio of normalized ARGs (or MGEs) to normalized 16S rRNA according to the method performed by Li et al. (2015). Briefly, the abundance of ARGs or MGEs was calculated as the following equation:

$$\text{Abundance}=\sum_{1}^{n}\frac{N_{\text{Gene}}\times L_{\text{reads}}/L_{\text{GeneRef}}}{N_{\text{16S \textbackslash{} rRNA}}\times L_{\text{reads}}/L_{\text{16S \textbackslash{} Ref}}},$$

where *N*_Gene_ is the reads number of the ARG or MGE; *L*_GeneRef_ is the reference sequence length of the corresponding ARG or MGE; *N*_16S rRNA_ is the reads number of the 16S rRNA; *L*_16S Ref_ is the average length of the 16S sequence in the Silva database; *n* is the number of the ARG or MGE genotype; *L*_reads_ is the sequence length of the Illumina reads (150 nt) in this study.

Before taxonomic classification, unigenes were annotated according to previous work (Wang et al., 2018) with two modifications: we used MEGANHIT v1.1 and SOAPaligner v2.21 with parameters “‐‐presets meta‐large” and “‐u, −2, ‐m 200,” respectively; we removed the genes that mapped reads less than or equal to 2 in the step “Gene prediction from scaftigs.” For taxonomic classification, the total unigenes were aligned against NCBI NR database (V20151204) using DIAMOND v0.9.14 (set as blastp, ‐e 1e‐5). The sequences with *e*‐value not more than the minimum *e*‐value × 10 were selected for the taxonomic annotation by LCA algorithm from MEGAN5 (Huson, Mitra, Ruscheweyh, Weber, & Schuster, 2011). Then, by combining the unigene abundance information, the abundance of all taxonomic levels in each sample can be calculated (Huson et al., 2011; Karlsson et al., 2013).

### 2.6 Statistical analysis

Bar charts were generated using Excel 2016 (Microsoft, USA). Venn diagrams were created online (http://bioinformatics.psb.ugent.be/webtools/Venn/). Difference between two groups was analyzed by SPSS v23.0 (IBM), statistical test is independent‐samples *T* test, Confidence Interval Percentage is 95%.

Spearman correlations coefficients were calculated by R3.4.3 with the Hmisc v4.1‐1 package. Correlation heatmaps were exported by Excel 2016 and PowerPoint 2016 (Microsoft). For accuracy of correlation analysis, the abundance of ARGs, resistance types, resistance mechanisms, MGEs and the classes, which covered at least four samples, were selected for analysis.

Principal component analysis (PCA), redundancy analysis (RDA), partial RDA (pRDA), Mantel test, and Procrustes test analysis were performed using R3.4.3 with the vegan v2.4‐6 package. Because the number of environmental factors exceeded the number of the samples, the first axis of MGEs PCA, the first two axes of sediment properties PCA and the first two axes of microbial community PCA were chosen for the RDA and pRDA similar to a previous study (Zhang et al., 2018). These axes account for 87.28%–99.90% of the variance in these factors. Network analysis was performed using Gephi v0.9.2 (Bastian, Heymann, & Jacomy, 2009). At the class level, *z*‐scores represent the standardized relative abundance of taxa were calculated by the following equation: $z-\text{score}=\frac{\left|\text{SG}-\text{TGA}\right|}{\mathrm{SD}},$ where SG is the relative abundance of individual taxa in a sample; TGA is the average relative abundance of the individual taxa in total samples; *SD* is the standard deviation of the individual taxa in total samples.

## 3 RESULTS

### 3.1 Abundance and diversity of ARGs, resistance type, and resistance mechanism

**Figure 2:**
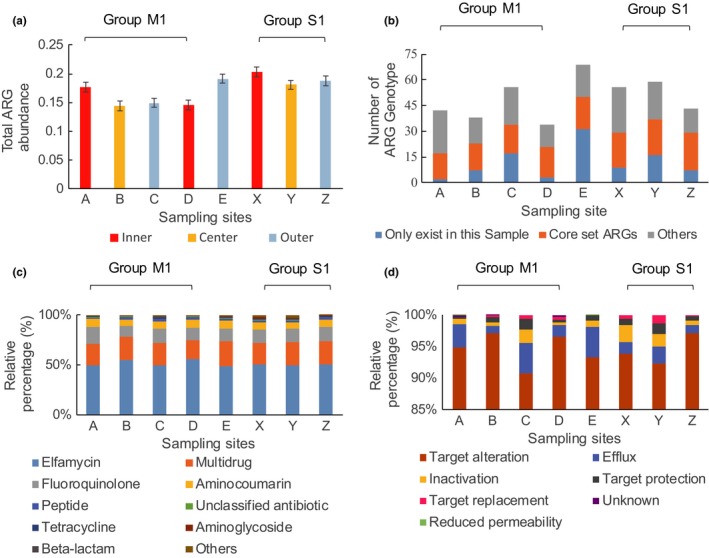
Profiles of antibiotic resistance genes (ARGs), resistance types, and resistance mechanisms. (a) Total abundance of ARGs, the difference color represents inner, center, or outer beach. (b) Number of ARG genotype, *y*‐axis represent the number of the ARGs genotypes. (c) Relative abundance of resistance types. (d) Relative abundance of resistance mechanisms

The abundance of the ARGs was described as the number of ARGs which normalized by 16S rRNA according to previous studies (Li et al., 2015). The abundances of ARGs in the samples were between 0.144 (sample B) and 0.203 (sample X), which are mangrove site and nonmangrove site, respectively (Figure 2a).

A total of 163 different ARGs were annotated (Table S1 and Figure 2b), and the genotypes of ARGs in each sample ranged from 34 (sample D) to 69 (sample E). ARGs were classified into 22 resistance types (Table S1) according to drug class. The abundances of the major resistance types were 0.703, 0.312, 0.186, 0.097, and 0.030 according to elfamycin, multidrug, fluoroquinolone, aminocoumarin, and peptide, respectively, and the abundances of these five resistance types were 96.39% of the total abundance (Figure 2c and Table S2).

Fifteen ARGs exist in all the samples (Figure 2b), which were called “core set ARGs.” These ARGs correspond to six resistance types, including five multidrug resistance genes *mexB* (Inner membrane multidrug exporter of the efflux complex MexAB‐OprM), *smeE* (Protein of the efflux complex SmeDEF), *rpoB* (Rifamycin‐resistant beta‐subunit of RNA polymerase), *rpsL* (Ribosomal protein S12), and *acrB* (Subunit of AcrA‐AcrB‐TolC multidrug efflux complex); four fluoroquinolone resistance genes *gyrA* (DNA gyrase subunit A), *parE* (DNA topoisomerase IV subunit B), *parC* (DNA topoisomerase IV subunit A), *mfd* (Transcription‐repair‐coupling factor); two peptide resistance genes *rpoC* (DNA‐directed RNA polymerase subunit beta) and *pmrE* (UDP‐glucose 6‐dehydrogenase); two aminocoumarin resistance genes *gyrB* (DNA gyrase subunit B), and *parY* (Subunit of topoisomerase IV); one elfamycin resistance gene *ef‐Tu* (Elongation factor Tu); and one unclassified ARG *katG* (Catalase‐peroxidase) (Figure 2b). Core set ARGs accounted for 94.44% of the total abundance of ARGs.

The following six of the seven resistance mechanisms in CARD were detected: antibiotic efflux, antibiotic inactivation, antibiotic target alteration, antibiotic target protection, antibiotic target replacement, and reduced permeability to antibiotics (Figure 2d). In addition, the term “unknown mechanism” was utilized for the ARG *tet34* (oxytetracycline resistance gene) (Table S1). Target alteration, efflux, inactivation, and target protection existed in all samples (Table S3) as major resistance mechanisms with 99.47% of the total abundance (Figure 2d).

### 3.2 Antibiotic resistance differences between mangrove and nonmangrove sediments

Excluding sample E, we divided all the samples into two groups: M1 and S1. Group M1 contained samples A, B, C, and D from the mangrove sediments of the north bank of the estuary. As a control group, S1 contained samples X, Y, and Z from the sandy beach (nonmangrove) on the south bank of the estuary.

**Figure 3:**
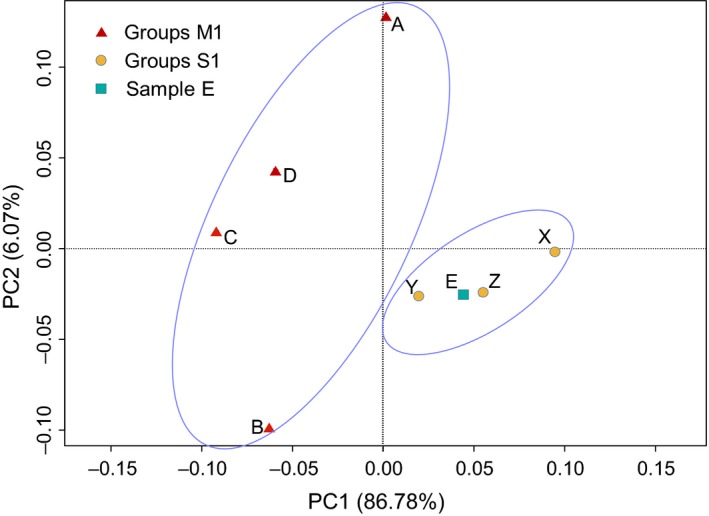
Principal component (PC) analysis plot resulting from differences of antibiotic resistance gene abundance of each sample

To investigate the differences of samples based on ARG compositional structure, PCA was employed. PCA plot (Figure 3) shows that samples of the two groups can be distinguished. It shows closer distances among samples of S1 group, whereas samples of M1 are more disperse. The Sample E was collected from the sandy beach outside the mangroves area, but it is closer to the nonmangrove samples on the PCA plot.

**Figure 4:**
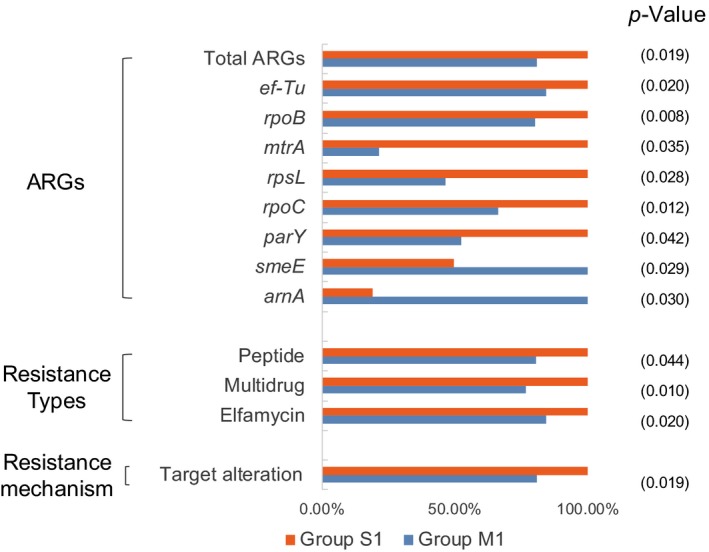
The relative abundance of antibiotic resistance genes (ARGs), resistance types and resistance mechanisms which was significant difference between group M1 and S1. And the more abundant variable is marked as 100%

**Figure A1:**
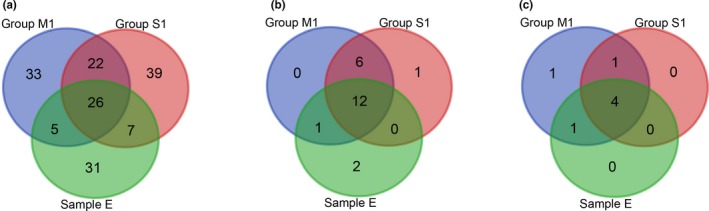
Venn diagrams for amounts of (a) antibiotic resistance gene genotypes, (b) resistance types, and (c) resistance mechanisms in groups M1, S1, and sample E

The average abundance of total ARGs in the group S1 was significantly greater (*p* < 0.05) than group M1. Eight ARGs, *mtrA* (transcriptional activator of the MtrCDE multidrug efflux pump),* arnA* (UDP‐4‐amino‐4‐deoxy‐L‐arabinose formyltransferase), *smeE*, *rpoB, rpoC*, *rpsL*,* ef‐Tu*, and *parY* were significantly different (*p* < 0.05) between M1 and S1 (Figure 4). There are 86 ARGs were found in M1 while 94 ARGs in S1, 48 ARGs existed both in the group M1 and S1, 33 ARGs were only detected in the group M1, and 39 only in the group S1 (Figure A1a).

Abundances of elfamycin, multidrug, and peptide‐resistant ARGs showed significant differences (*p* < 0.05) between the two groups, and they are higher in S1 (Figure 4). The Venn diagram analysis (Figure A1b) shows that group M1 and S1 shared most resistance types.

Only one resistance mechanism, target alteration, showed significant difference between group M1 and S1 (*p* < 0.05) (Figure 4). Group M1 contained all the five resistance mechanisms that found in S1, and had unique unknown mechanism (Table S3 and Figure A1c).

### 3.3 Sediment properties

**Figure 5:**
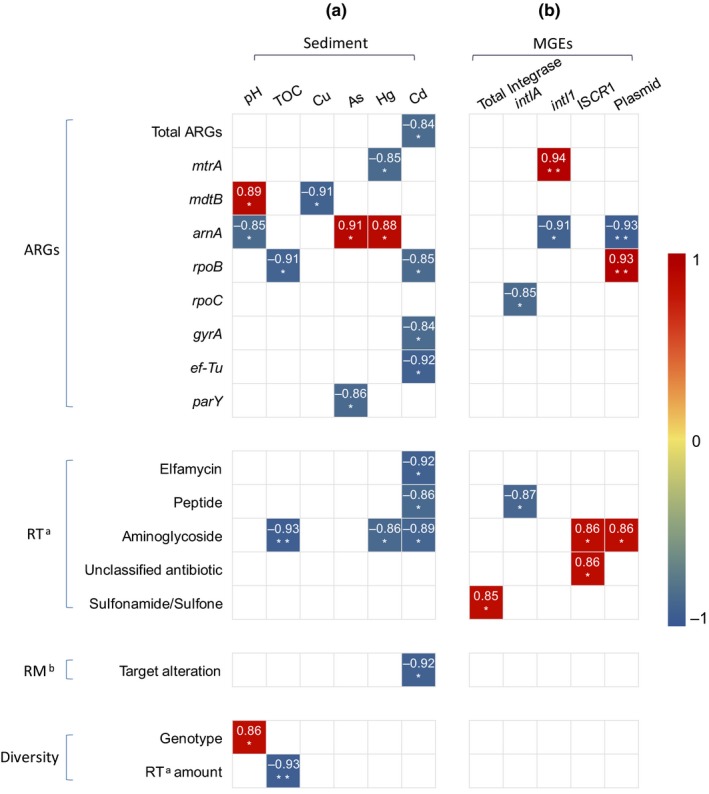
Significant correlation between (a) sediment properties and antibiotic resistance variables (ARVs) and (b) mobile genetic elements (MGEs) and ARVs. ^a^RT, resistance type; ^b^RM, resistance mechanism. *p < 0.05; **p < 0.01; ***p < 0.001

To study the effects of sediment properties on antibiotic resistance, the pH, TOC, heavy metal (Cu, Zn, Hg, Cd) and metalloid As contents of the samples were determined (Table S4). The average pH and TOC of M1 group were both higher than S1 group, and the difference of TOC was significant (*p* < 0.05). Zn was the highest metal (metalloid) with a content up to 52.9 mg/kg in the sample X. The contents of As, Hg, and Cd were significantly higher in M1 group than that in S1 group (*p* < 0.05). Figure 5 shows the significant correlations (*p* < 0.01) between sediment properties and antibiotic resistance variables (ARVs) consisting of the abundances and diversity of ARGs, resistance types and resistance mechanisms. The significant negative correlations between sediment properties and ARVs were found by correlations analysis (*p* < 0.01).

Metal Cd was the most frequent factor related to ARVs. It negatively correlated to total ARG abundance, abundance of ARGs *rpoB*, *gyrA,* and* ef*‐*Tu*, abundance of resistance type elfamycin, peptide and aminoglycoside, and abundance of resistance mechanism target alteration. The pH and TOC showed significant (*p* < 0.01) correlation with ARG and resistance type of ARVs. Similar correlations were found in Cu, As, and Hg. No significant (*p* < 0.01) correlation was found between Zn and ARVs.

### 3.4 MGES

The integrase genes, ISCR, and plasmids that are potentially related to HGT were annotated, as shown in Table 1. These sequences represented the MGEs in the samples. The high coverage genes *intI* (unclassified integron integrase gene in the INTEGRALL database), *intI1*, *intIA*, and IS*CR1* were detected in the most samples, and the abundance of plasmids in each sample reached 7.27–13.36.

**Table 1:** Abundance of mobile genetic elements (MGEs) in each sample (ppm)

MGEs	Mangrove area (M1)					Nonmangrove area (S1)		
	A	B	C	D	E	X	Y	Z
*intI*	4.91E−03	6.85E−03	2.57E−03	3.10E−03	4.46E−03	5.10E−03	4.93E−03	4.37E−03
*intI1*	0.00E + 00	0.00E + 00	2.49E−04	0.00E + 00	0.00E + 00	1.35E−03	2.19E−03	3.23E−04
*intI2*	1.07E−04	0.00E + 00	8.63E−05	0.00E + 00	0.00E + 00	0.00E + 00	0.00E + 00	0.00E + 00
*intI3*	0.00E + 00	2.08E−04	0.00E + 00	0.00E + 00	0.00E + 00	3.22E−04	0.00E + 00	0.00E + 00
*intI4*	0.00E + 00	1.06E−04	0.00E + 00	0.00E + 00	0.00E + 00	0.00E + 00	0.00E + 00	0.00E + 00
*intIA*	1.06E−03	7.03E−04	2.79E−04	6.51E−04	7.89E−04	0.00E + 00	1.20E−04	0.00E + 00
IS*CR1*	4.71E−04	1.98E−04	1.63E−04	3.05E−04	6.23E−04	1.01E−03	9.25E−04	4.24E−04
IS*CR14*	0.00E + 00	0.00E + 00	0.00E + 00	0.00E + 00	0.00E + 00	1.50E−04	3.69E−04	7.34E−05
Plasmid	9.67E + 00	7.80E + 00	9.77E + 00	7.27E + 00	1.02E + 01	1.11E + 01	1.13E + 01	1.34E + 01

Note

Genes* intI1*, *intI2*, *intI3*, *intI4*, and *intIA* are the different types of integron integrase gene, and the *intI* is the unclassified integron integrase gene in the INTEGRALL database. IS*CR*1 and IS*CR*14 are the different types of insertion sequence common region.

In the MGEs, *intIA* and plasmid showed significant difference (*p* < 0.05) between M1 and S1. The average abundance of plasmid in the group S1 was significantly greater (*p* < 0.05) than group M1, while *intIA* was different.

Figure 5 shows the significant correlations (*p* < 0.01) between MGEs and ARVs. The abundance of total integrase genes correlated to resistance type sulfonamide/sulfone, IS*CR*1 and plasmid mainly showed positive correlation with the ARVs, while *intIA* was showed only negative correlation with the ARVs. No significant (*p* < 0.01) correlation were found between *intI* and ARVs.

### 3.5 Microbial community

To research the effect of microbial community on the distribution of ARGs, a total of 93 classes were identified in the samples. Fifty classes showed significant (*p* < 0.01) correlations with at least one variable of ARVs, resulting in a total of 111 correlations that contained 57 positive correlations and 54 negative correlations.

**Figure 6:**
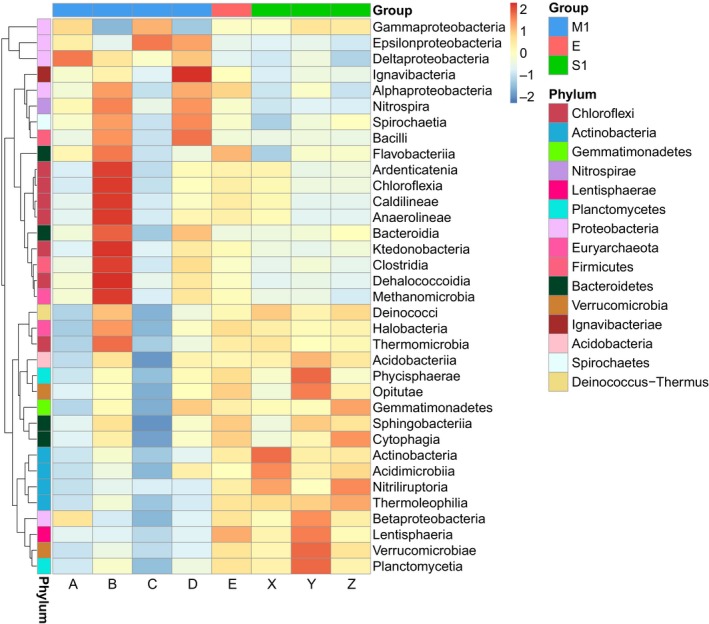
Abundance heatmap according to *z*‐scores of the most abundant 35 classes

To investigate the major change of microbial community between M1 and S1, we chose the most abundant 35 classes from all the 93 classes that accounted for more than 98.96% of total abundance in each sample and represented the dominant classes. Figure 6 shows the abundances of the most abundant 35 classes, which belong to 15 phyla. About half of the classes were more abundant in samples of M1 than in S1, while the others were different.

### 3.6 Correlation among ARGS, sediment properties and MGES, as well as microbial community

**Figure 7:**
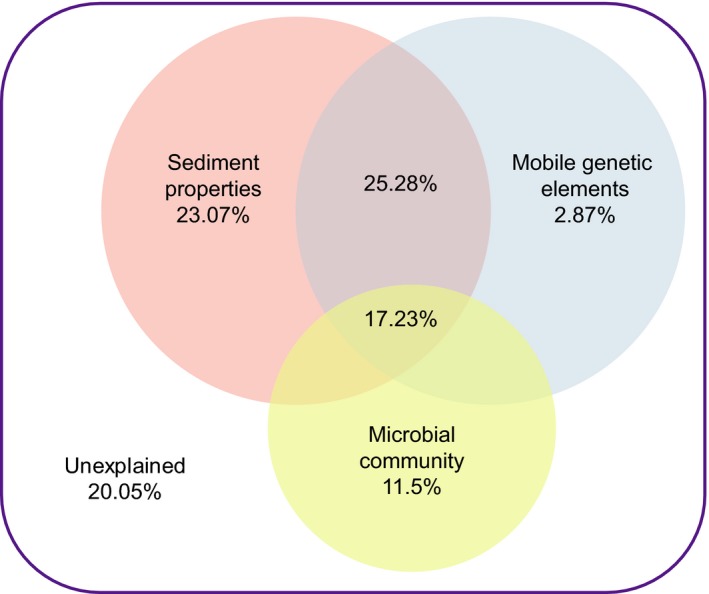
Venn analysis for the contribution of microbial community, sediment properties, and mobile genetic elements to variation of antibiotic resistance gene

The results of pRDA showed that the microbial community, sediment properties, and MGEs explained 79.95% of the differences in the distribution of ARGs (Figure 7). Sediment properties explained 23.07% alone, MGEs explained 2.87% alone, and they collectively explained 51.22%, while the microbial community contributed 11.50% alone.

For sediment properties and microbial community, the two major factors contributed to the ARG distribution alone, the Mantel test showed a matrix‐level significant correlation between the ARGs and sediment properties (Spearman's *r* = 0.409, *p* = 0.016, Euclidean distance, permutations: 9,999), and that of microbial community (Spearman's *r* = 0.382, *p* = 0.038, Euclidean distance, permutations: 9,999). The results of Procrustes test showed that the similarity of PCA results between ARGs and microbial community were significant (*M* ^2^ = 0.520, Spearman's *r* = 0.693, *p* = 0.047, permutations: 9,999).

## 4 DISCUSSION

### 4.1 Impact of mangroves as natural wetland on ARGs

The estuarine water flow is complex, and the sediments are periodically soaked by tides, making it difficult to research straightforwardly the impact of mangrove area on ARGs through the analysis of ARG abundance in influents and effluents, unlike in sewage treatment plants and constructed wetlands (Chen et al., 2015; Yang, Li, Zou, Fang, & Zhang, 2014). However, we achieved this aim by comparing ARVs between mangrove and nonmangrove sediments, which were considered to receive identical pollutions in the same estuary.

For the abundance, the most abundant ARGs and the total ARGs, the most abundant resistance types, and the most resistance mechanism in mangrove sediment were significantly lower than that in nonmangrove sediment in this study (*p* < 0.05) (Figure 4). The results indicated that mangrove ecosystems have the capacity to remove ARGs as a natural wetland. Recently research indicates that antibiotics used in aquaculture were commonly occurred in mangroves, and the antibiotic pollutions can be mitigated with mangrove vegetation to some extent (Li et al., 2016). The lack of selective pressure from antibiotics may contribute to the ARGs reducing, because antibiotic resistance always carries a fitness cost (Vogwill & MacLean, 2015). Biodegradation is considered to be one of the reasons for removing ARGs in the integrated constructed wetland (Chen et al., 2015), and anaerobic digestion can effectively remove ARGs during swine manure treatment (Zhang et al., 2018). And we found that the sediment properties were much different between the M1 and S1 groups, such as TOC, As, Hg, and Cd were significantly lower in M1 than that in S1 (Table S4). The sediment properties (pH, TOC, Cu, As, Hg, and Cd) were significantly correlated to ARGs such as *mdtB* (subunit of the MdtABC‐TolC efflux complex), *arnA*, and *rpoB* (Figure 5). In addition, the pRDA results showed the contribution of the sediment properties and microbial community to ARG distribution (Figure 7). It is indicated that the sediment properties and bacterium in mangrove sediment may contribute to the elimination of ARGs.

To gain deep insight into the fate of ARGs in mangrove environments, further researches in different types of mangrove ecosystems are necessary. Because the pattern of mangroves impact on ARGs may be different. For example, studies of ARGs in constructed wetlands show variant results. Some studies on constructed wetlands observed the efficient removal of ARGs (Chen et al., 2015; Fang et al., 2017), while others only observed a slight effect (Berglund et al., 2014; Hsu, Mitsch, Martin, & Lee, 2017). Regardless, our work may serve as a precedent for ARG research in the mangrove ecosystem.

### 4.2 Correlations between antibiotic resistance and abiotic factors, as well as biological factors

To further understand the potential factors that influence the antibiotic resistance, we analyzed the relationship between ARVs and abiotic factors (properties of sediment), as well as biological factors (microbial community and MGEs) in the samples.

Metals such as lead, copper, zinc, cadmium, and metalloid arsenic are often used in agriculture and aquaculture (Singer, Shaw, Rhodes, & Hart, 2016). Since resistance to antibiotics and metals can share certain mechanisms (Singer et al., 2016), the phenomenon called coselection between them has been reported, and the stress of widespread metals in the environment may promote this phenomenon (Li, Xia, & Zhang, 2017). Especially, Cu and Zn that were reported to have an extensive positive correlation with antibiotic resistance (Ji et al., 2012; Wang et al., 2016; Zhu et al., 2013) in the samples from livestock farms. However, in the total metal or metalloid elements detected in this study, only As and Hg positively correlated to abundance of *arnA*. While the others showed negative correlation to the different ARVs, except zinc has no correlation with ARVs (Figure 5). The previous study showed that the occurrence of coselection dependent on whether the metal concentration reaches the minimum coselective concentration of an environment (Seiler & Berendonk, 2012). The metal concentrations in this study are natural background concentrations (Ministry of Ecology & Environment of the People's Republic of China, 1995). And the concentrations of Cu and Zn are much lower than the samples from livestock farms in the above study (Ji et al., 2012; Wang et al., 2016; Zhu et al., 2013). In our study, sediment TOC was found to negatively correlate to gene *rpoB*, resistance type aminoglycoside and amount of resistance types (Figure 5), and pH was positively correlated to abundance of gene *mdtB* and ARG diversity, and negatively correlated to abundance of gene *arnA*. Correlations between ARGs and pH or TOC in different soil researches are variant (Knapp et al., 2011; Wang et al., 2016), it seems that TOC and pH affect ARGs depends on the specific environmental background.

Many studies showed that MGEs, including ISCR, plasmids, transposases, and integrons, have a significant role in promoting HGT of ARGs (Ouyang et al., 2015; Zhang et al., 2016). Positive correlations between MGEs and ARVs were found in our study, including plasmid, total abundance of integrase, class one integrase *intI1*, and IS*CR1* (Figure 5). However, *intIA* showed only negative correlation with two ARVs (*p* < 0.05) (Figure 5).

**Figure A2:**
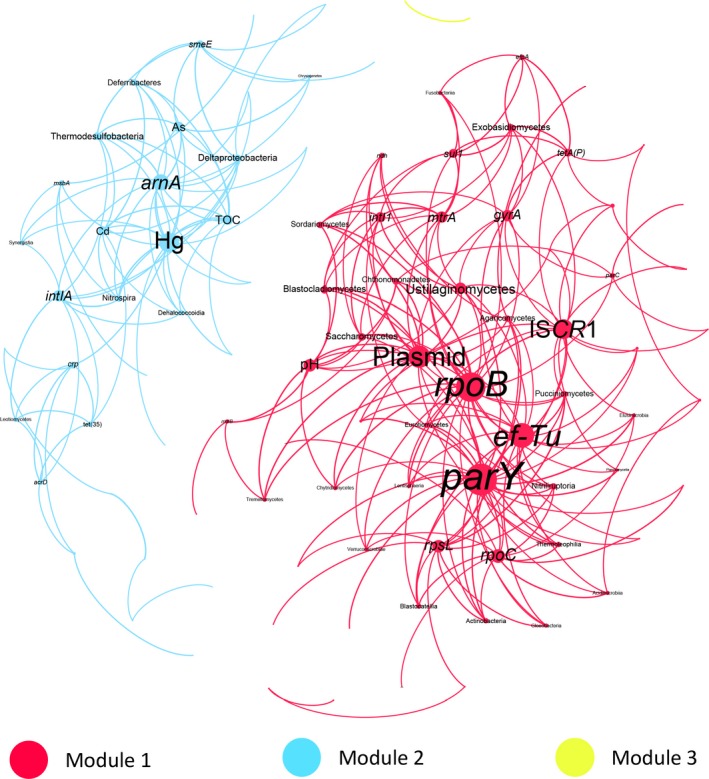
Network analysis of cooccurrence of antibiotic resistance genes and mobile genetic elements with sediment properties and microbial community. The following nodes are labeled: nodes that connect at least five other nodes and the core nodes of the module. The font size is displayed according to the degree of connections; a larger font size indicates more connections

In this study, results of pRDA showed that the sediment properties and MGEs were the main causes for the differences in the distribution of ARGs, and the sediment properties is the most influential factor for ARG distribution rather than microbial community. We also analyzed the cooccurrence pattern among ARGs, MGEs, sediment properties, and microbial community using network analysis (Spearman's *r* > 0.7, *p* < 0.05). The network was divided into two major modules and one mini module after modularity analysis (Figure A2). MGEs including plasmid, IS*CR*1 and *intI1* located at the key nodes of the module one, indicating that these MGEs play an essential role in the module. And the sediment properties such as Hg, TOC, Cd, and As were located at the key nodes of the module two, these sediment factors may affect the ARG distribution in the module. This is similar to the results of studies on Chinese estuaries at a continental scale (Zhu et al., 2017). However, another research on sewage treatment process showed that bacterial community and MGEs are the main factors impacting the fate of ARGs (Zhang et al., 2016, 2018). This suggested that the most influential factors affecting the ARG distribution are different in the natural environment and biological treatment processing, but the MGEs have a substantial impact on ARGs. The network analysis also confirmed that a variety of ARGs and MGEs connected to the same class, this indicated the possibility of the ARGs migrating in the modules through HGT, which may lead to the emergence of antibiotic‐resistant strains.

### 4.3 Profiles of antibiotic resistance in the subtropical urban estuary adjacent to the south china sea

It was reported that ARG abundance range from 0.066 to 0.543 among 18 Chinese estuaries (Zhu et al., 2017). The average ARG abundance in our study is 0.170, which is in the same order of magnitude as a previous survey (Zhu et al., 2017). It seems that this abundance range is the current situation of Chinese estuaries. However, it is much lower than the ARG abundances of samples from urban sewage treatment plants influents and wastewater from livestock farm (Zhu et al., 2013).

Twenty‐two antibiotic resistance types were found in this work. The most abundant three resistance types in each sample are elfamycin, multidrug, and fluoroquinolone (Figure 2). Elfamycin resistance in the study was mainly contributed by the most abundant ARG *ef‐Tu* which is a prokaryotic elongation factor gene that becomes ARG due to mutation (Cappellano, Monti, Sosio, Donadio, & Sarubbi, 1997; Prezioso, Brown, & Goldberg, 2017). Multidrug resistance is mainly contributed by the second highest abundance ARG *rpoB* and vast efflux genes. The *rpoB* is also an evolved ARG from RNA polymerase gene, were widely found in pathogenic mycobacteria (Andre et al., 2017). The majority of efflux pumps are encoded by chromosomes (Blanco et al., 2016) and considered as a general metabolic mechanism (Borges‐Walmsley, Mckeegan, & Walmsley, 2003). This coincided with a point of view that lots of ARGs evolved from natively functional genes of the bacterium (Wright, 2007). Fluoroquinolone is the most used antibiotic in China and the fluoroquinolone resistant genes were prevalent in China (Xu et al., 2009; Zhang et al., 2015; Zhang, Lu, Wang, Pang, & Zhao, 2014), similar situation was found in several relatively abundant resistance types such as β‐lactam and tetracycline (Wang et al., 2017; ZHANG et al., 2017, 2015; Zhu et al., 2017).

## 5 CONCLUSION

In this study, profiles of ARGs in a mangrove area at an urban estuary in the South China Sea were investigated. The abundance of ARGs was significantly lower in mangrove sediment than that in nonmangrove sediment (*p* < 0.05), suggesting the ARGs‐removed capacity of the subtropical mangrove wetland ecosystems. And sediment properties together with MGEs were the most important factors impacting the distribution of ARGs rather than microbial community.

## CONFLICT OF INTERESTS

All authors declare no competing interests.

## AUTHOR CONTRIBUTIONS

HZ, CJ, and BW have set up and designed the study; HZ, BY, and XM have set up and collected samples; SM and QO have performed the experiments of DNA extraction and sediments properties determination; HZ, PL, and NL have analyzed the data; HZ and CJ have written the manuscript and BL, QL, and PS have revised it. All authors discussed the results and commented the manuscript and all authors read and approved the final manuscript.

## ETHICS STATEMENT

The study was carried out under the stipulation of ethics approved by the Guangxi Key Lab of Mangrove Conservation and Utilization, Guangxi Mangrove Research Center, Guangxi Academy of Sciences; the State Key Laboratory for Conservation and Utilization of Subtropical Agro‐bioresources, College of Life Science and Technology, Guangxi University.

## DATA ACCESSIBILITY

All the metagenomic sequencing data analyzed in this work were deposited in the NCBI SRA database under the BioProject PRJNA472209.
