# Supplementary material for: Prevalence and proliferation of antibiotic resistance genes in the subtropical mangrove wetland ecosystem of South China Sea
Source: Microbiologyopen. 2019 Jun 28;8(11):e871. doi: 10.1002/mbo3.871 (PMC6855136; doi:10.1002/mbo3.871)
Supplement: Supplementary file 1 [file MBO3-8-e871-s001.docx]

Prevalence and Proliferation of Antibiotic Resistance Genes in the Subtropical Mangrove Wetland Ecosystem of South China Sea

**Huaxian Zhao^1, 2, 3^, Bing Yan^1, 2^, Xueyan Mo^3^, Pu Li^4^, Baoqin Li^6^, Quanwen Li^3^, Nan Li^5^, Shuming Mo^3^, Qian Ou^3^, Peihong Shen^3^, Bo Wu^7*^, and Chengjian Jiang^1, 2, 3**^**

^1^ Guangxi Key Lab of Mangrove Conservation and Utilization, Guangxi Mangrove Research Center, Guangxi Academy of Sciences, Beihai 536000, China

^2^ Guangxi Key Laboratory of Marine Natural Products and Combinatorial Biosynthesis Chemistry, Guangxi Academy of Sciences, 98 Daling Road, Nanning 530007, China.

^3^ State Key Laboratory for Conservation and Utilization of Subtropical Agro-bioresources, College of Life Science and Technology, Guangxi University, 100 Daxue East Road, Nanning, Guangxi, 530004, China.

^4^ PFOMIC Bioinformatics Company, 9 Huoju Road, Nanning, Guangxi, 530004, China.

^5^ Key Laboratory of Environment Change and Resources Use in Beibu Gulf, Ministry of Education (Nanning Normal University), 175 Mingxiu East Road, Nanning, Guangxi, 530001, China.

^6^ Guangdong Key Laboratory of Integrated Agro-environmental Pollution Control and Management, Guangdong Institute of Eco-Environmental Science & Technology, 808 Tianyuan Road, Guangzhou, 510650, China.

^7^ Department of chemical and biological engineering, Guangxi Normal University for Nationalities, 1 Lichuan Road, Chongzuo, 532200, China.

* and ** : Corresponding Author:

* Bo Wu:

Tel: +86-771-3270736; Fax: +86-771-3237873

657575168@qq.com

** Chengjian Jiang:

Tel: +86-771-3270736; Fax: +86-771-3237873

[jiangcj0520@gmail.com](mailto:jiangcj0520@gmail.com)

**Supplementary Table S1.** Annotation and abundance of detected ARGs.

| ARO | Annotation | Resistance type | Resistance mechanism | Mangrove area | | | |  | Non-mangrove area | | |
| --- | --- | --- | --- | --- | --- | --- | --- | --- | --- | --- | --- |
|  |  |  |  | A | B | C | D | E | X | Y | Z |
| 3000781 | *adeJ* | Multidrug | Efflux | 0.00E+00 | 0.00E+00 | 0.00E+00 | 0.00E+00 | 0.00E+00 | 0.00E+00 | 3.45E-05 | 0.00E+00 |
| 3000167 | *tet(C)* | Tetracycline | Efflux | 0.00E+00 | 0.00E+00 | 0.00E+00 | 0.00E+00 | 0.00E+00 | 0.00E+00 | 1.84E-04 | 0.00E+00 |
| 3002703 | *cmx* | Phenicol | Efflux | 0.00E+00 | 0.00E+00 | 0.00E+00 | 0.00E+00 | 0.00E+00 | 0.00E+00 | 3.72E-04 | 0.00E+00 |
| 3000196 | *tet32* | Tetracycline | Target Protection | 0.00E+00 | 0.00E+00 | 0.00E+00 | 0.00E+00 | 3.12E-04 | 1.73E-04 | 0.00E+00 | 0.00E+00 |
| 3000074 | *emrB* | Fluoroquinolone | Efflux | 0.00E+00 | 0.00E+00 | 0.00E+00 | 0.00E+00 | 3.90E-04 | 0.00E+00 | 0.00E+00 | 0.00E+00 |
| 3000778 | *adeG* | Multidrug | Efflux | 0.00E+00 | 0.00E+00 | 1.37E-04 | 0.00E+00 | 0.00E+00 | 0.00E+00 | 0.00E+00 | 0.00E+00 |
| 3000180 | *tetA(P)* | Tetracycline | Efflux | 3.28E-04 | 0.00E+00 | 0.00E+00 | 0.00E+00 | 0.00E+00 | 7.90E-04 | 1.73E-04 | 8.61E-05 |
| 3002839 | *lnuF* | Lincosamide | Inactivation | 1.26E-04 | 0.00E+00 | 0.00E+00 | 0.00E+00 | 0.00E+00 | 1.35E-04 | 0.00E+00 | 0.00E+00 |
| 3000481 | *tet(35)* | Tetracycline | Efflux | 2.80E-04 | 2.75E-04 | 7.55E-04 | 1.69E-04 | 4.32E-04 | 0.00E+00 | 0.00E+00 | 0.00E+00 |
| 3000254 | *emrY* | Tetracycline | Efflux | 0.00E+00 | 0.00E+00 | 0.00E+00 | 0.00E+00 | 7.79E-05 | 0.00E+00 | 0.00E+00 | 0.00E+00 |
| 3000205 | *tetX* | Tetracycline | Inactivation | 0.00E+00 | 0.00E+00 | 0.00E+00 | 0.00E+00 | 0.00E+00 | 0.00E+00 | 0.00E+00 | 9.32E-05 |
| 3002524 | *aac(2')-Ib* | Aminoglycoside | Inactivation | 1.76E-04 | 0.00E+00 | 0.00E+00 | 0.00E+00 | 0.00E+00 | 0.00E+00 | 0.00E+00 | 0.00E+00 |
| 3002837 | *lnuC* | Lincosamide | Inactivation | 0.00E+00 | 0.00E+00 | 0.00E+00 | 0.00E+00 | 0.00E+00 | 6.72E-04 | 2.21E-04 | 0.00E+00 |
| 3000816 | *mtrA* | Multidrug | Efflux | 3.01E-04 | 0.00E+00 | 6.10E-04 | 0.00E+00 | 0.00E+00 | 6.46E-04 | 1.59E-03 | 9.50E-04 |
| 3000378 | *mexB* | Multidrug | Efflux | 3.96E-04 | 3.24E-05 | 3.74E-04 | 1.20E-04 | 3.82E-05 | 1.41E-04 | 6.97E-05 | 3.46E-05 |
| 3002615 | *aadA15* | Aminoglycoside | Inactivation | 0.00E+00 | 0.00E+00 | 0.00E+00 | 0.00E+00 | 0.00E+00 | 2.80E-04 | 0.00E+00 | 0.00E+00 |
| 3000478 | *tet(33)* | Tetracycline | Efflux | 0.00E+00 | 0.00E+00 | 0.00E+00 | 0.00E+00 | 0.00E+00 | 0.00E+00 | 0.00E+00 | 1.78E-04 |
| 3003010 | *ceoB* | Multidrug | Efflux | 0.00E+00 | 0.00E+00 | 0.00E+00 | 0.00E+00 | 0.00E+00 | 3.60E-05 | 0.00E+00 | 3.53E-05 |
| 3002791 | *qnrS2* | Fluoroquinolone | Target Protection | 0.00E+00 | 0.00E+00 | 2.55E-04 | 0.00E+00 | 0.00E+00 | 0.00E+00 | 0.00E+00 | 0.00E+00 |
| 3003109 | *msrE* | Multidrug | Efflux | 0.00E+00 | 0.00E+00 | 0.00E+00 | 0.00E+00 | 0.00E+00 | 0.00E+00 | 4.45E-04 | 7.37E-05 |
| 3000808 | *mexI* | Multidrug | Efflux | 0.00E+00 | 0.00E+00 | 1.63E-04 | 0.00E+00 | 0.00E+00 | 0.00E+00 | 0.00E+00 | 0.00E+00 |
| 3000206 | *emrK* | Tetracycline | Efflux | 0.00E+00 | 0.00E+00 | 0.00E+00 | 0.00E+00 | 2.27E-04 | 0.00E+00 | 0.00E+00 | 0.00E+00 |
| 3002792 | *qnrS3* | Fluoroquinolone | Target Protection | 0.00E+00 | 1.55E-04 | 0.00E+00 | 0.00E+00 | 0.00E+00 | 0.00E+00 | 0.00E+00 | 0.00E+00 |
| 3000833 | *evgS* | Multidrug | Efflux | 0.00E+00 | 0.00E+00 | 2.33E-05 | 0.00E+00 | 1.33E-04 | 0.00E+00 | 0.00E+00 | 0.00E+00 |
| 3000361 | *ereA* | Macrolide | Inactivation | 0.00E+00 | 0.00E+00 | 0.00E+00 | 0.00E+00 | 0.00E+00 | 5.45E-04 | 0.00E+00 | 0.00E+00 |
| 3001214 | *mdtM* | Multidrug | Efflux | 0.00E+00 | 0.00E+00 | 0.00E+00 | 0.00E+00 | 9.73E-05 | 0.00E+00 | 0.00E+00 | 0.00E+00 |
| 3000793 | *mdtB* | Aminocoumarin | Efflux | 0.00E+00 | 0.00E+00 | 2.69E-05 | 0.00E+00 | 1.92E-04 | 0.00E+00 | 3.51E-05 | 3.48E-05 |
| 3002826 | *ereA2* | Macrolide | Inactivation | 0.00E+00 | 0.00E+00 | 0.00E+00 | 0.00E+00 | 0.00E+00 | 0.00E+00 | 1.78E-04 | 0.00E+00 |
| 3002655 | *aph(4)-Ia* | Aminoglycoside | Inactivation | 0.00E+00 | 0.00E+00 | 0.00E+00 | 0.00E+00 | 0.00E+00 | 2.16E-04 | 0.00E+00 | 0.00E+00 |
| 3002647 | *aph(3')-IIIa* | Aminoglycoside | Inactivation | 2.61E-04 | 0.00E+00 | 0.00E+00 | 0.00E+00 | 1.51E-04 | 0.00E+00 | 0.00E+00 | 5.47E-04 |
| 3000174 | *tet(G)* | Tetracycline | Efflux | 0.00E+00 | 0.00E+00 | 0.00E+00 | 0.00E+00 | 0.00E+00 | 2.83E-04 | 0.00E+00 | 0.00E+00 |
| 3000616 | *mel* | Macrolide | Efflux | 0.00E+00 | 0.00E+00 | 0.00E+00 | 6.41E-05 | 0.00E+00 | 0.00E+00 | 0.00E+00 | 0.00E+00 |
| 3002794 | *qnrS5* | Fluoroquinolone | Target Protection | 0.00E+00 | 1.55E-04 | 0.00E+00 | 0.00E+00 | 0.00E+00 | 0.00E+00 | 0.00E+00 | 0.00E+00 |
| 3000794 | *mdtC* | Aminocoumarin | Efflux | 0.00E+00 | 0.00E+00 | 0.00E+00 | 0.00E+00 | 7.79E-05 | 0.00E+00 | 3.56E-05 | 3.53E-05 |
| 3000803 | *mexE* | Multidrug | Efflux | 0.00E+00 | 0.00E+00 | 2.02E-04 | 0.00E+00 | 0.00E+00 | 0.00E+00 | 0.00E+00 | 0.00E+00 |
| 3002641 | *aph(3')-Ia* | Aminoglycoside | Inactivation | 0.00E+00 | 0.00E+00 | 0.00E+00 | 0.00E+00 | 0.00E+00 | 0.00E+00 | 2.68E-04 | 0.00E+00 |
| 3004108 | *rob* | Multidrug | Efflux | 0.00E+00 | 0.00E+00 | 0.00E+00 | 0.00E+00 | 5.51E-04 | 0.00E+00 | 0.00E+00 | 0.00E+00 |
| 3002812 | *pp-flo* | Phenicol | Efflux | 0.00E+00 | 0.00E+00 | 0.00E+00 | 0.00E+00 | 2.13E-04 | 0.00E+00 | 0.00E+00 | 0.00E+00 |
| 3000796 | *mdtF* | Multidrug | Efflux | 0.00E+00 | 0.00E+00 | 0.00E+00 | 3.01E-05 | 3.85E-05 | 0.00E+00 | 3.52E-05 | 0.00E+00 |
| 3002597 | *aac(6')-Ie-APH(2'')-Ia* | Aminoglycoside | Inactivation | 0.00E+00 | 0.00E+00 | 0.00E+00 | 0.00E+00 | 3.33E-04 | 0.00E+00 | 0.00E+00 | 0.00E+00 |
| 3000805 | *oprN* | Multidrug | Efflux | 0.00E+00 | 0.00E+00 | 5.91E-05 | 0.00E+00 | 0.00E+00 | 0.00E+00 | 0.00E+00 | 0.00E+00 |
| 3000375 | *ermB* | MLS | Target Alteration | 0.00E+00 | 0.00E+00 | 0.00E+00 | 0.00E+00 | 1.61E-04 | 0.00E+00 | 0.00E+00 | 0.00E+00 |
| 3000499 | *acrE* | Multidrug | Efflux | 0.00E+00 | 0.00E+00 | 0.00E+00 | 0.00E+00 | 2.07E-04 | 0.00E+00 | 0.00E+00 | 0.00E+00 |
| 3000801 | *mexD* | Multidrug | Efflux | 0.00E+00 | 0.00E+00 | 0.00E+00 | 0.00E+00 | 0.00E+00 | 0.00E+00 | 3.50E-05 | 0.00E+00 |
| 3000410 | *sul1* | Sulfonamide, sulfone | Target Replacement | 1.23E-04 | 1.21E-04 | 0.00E+00 | 0.00E+00 | 0.00E+00 | 5.28E-04 | 1.69E-03 | 1.29E-04 |
| 3002985 | *arnA* | Peptide | Target Alteration | 4.69E-04 | 3.58E-04 | 1.69E-04 | 5.66E-04 | 3.01E-04 | 1.12E-04 | 0.00E+00 | 1.09E-04 |
| 3000245 | *rbpA* | Rifamycin | Target Protection | 0.00E+00 | 0.00E+00 | 2.43E-04 | 0.00E+00 | 0.00E+00 | 0.00E+00 | 0.00E+00 | 0.00E+00 |
| 3003066 | *smeR* | Multidrug | Efflux | 1.05E-03 | 0.00E+00 | 4.86E-04 | 4.08E-04 | 0.00E+00 | 0.00E+00 | 3.17E-04 | 4.73E-04 |
| 3000804 | *mexF* | Multidrug | Efflux | 3.25E-05 | 0.00E+00 | 6.31E-04 | 0.00E+00 | 0.00E+00 | 3.48E-05 | 3.43E-05 | 0.00E+00 |
| 3002681 | *catB9* | Phenicol | Inactivation | 1.64E-04 | 1.61E-04 | 0.00E+00 | 1.49E-04 | 0.00E+00 | 0.00E+00 | 0.00E+00 | 0.00E+00 |
| 3002707 | *qnrA1* | Fluoroquinolone | Target Protection | 0.00E+00 | 0.00E+00 | 1.28E-04 | 0.00E+00 | 0.00E+00 | 0.00E+00 | 0.00E+00 | 0.00E+00 |
| 3000556 | *tet44* | Tetracycline | Target Protection | 0.00E+00 | 0.00E+00 | 0.00E+00 | 0.00E+00 | 0.00E+00 | 2.88E-04 | 5.69E-05 | 0.00E+00 |
| 3000027 | *emrA* | Fluoroquinolone | Efflux | 0.00E+00 | 0.00E+00 | 0.00E+00 | 0.00E+00 | 1.02E-04 | 0.00E+00 | 0.00E+00 | 0.00E+00 |
| 3002897 | *sat-4* | Nucleoside | Inactivation | 0.00E+00 | 0.00E+00 | 0.00E+00 | 0.00E+00 | 2.21E-04 | 0.00E+00 | 0.00E+00 | 0.00E+00 |
| 3000518 | *crp* | Multidrug | Efflux | 1.80E-03 | 4.82E-04 | 1.59E-03 | 7.42E-04 | 1.33E-03 | 7.01E-04 | 0.00E+00 | 0.00E+00 |
| 3002660 | *aph(6)-Id* | Aminoglycoside | Inactivation | 0.00E+00 | 0.00E+00 | 0.00E+00 | 0.00E+00 | 0.00E+00 | 7.95E-04 | 2.62E-04 | 0.00E+00 |
| 3002605 | *aadA5* | Aminoglycoside | Inactivation | 0.00E+00 | 0.00E+00 | 0.00E+00 | 0.00E+00 | 0.00E+00 | 0.00E+00 | 5.55E-04 | 0.00E+00 |
| 3000195 | *tetB(P)* | Tetracycline | Target Protection | 0.00E+00 | 1.04E-04 | 0.00E+00 | 0.00E+00 | 0.00E+00 | 3.40E-04 | 0.00E+00 | 2.78E-04 |
| 3000191 | *tetQ* | Tetracycline | Target Protection | 0.00E+00 | 0.00E+00 | 0.00E+00 | 0.00E+00 | 6.07E-05 | 0.00E+00 | 0.00E+00 | 0.00E+00 |
| 3002704 | *fexA* | Phenicol | Efflux | 0.00E+00 | 0.00E+00 | 0.00E+00 | 0.00E+00 | 0.00E+00 | 0.00E+00 | 0.00E+00 | 7.62E-05 |
| 3002709 | *qnrA3* | Fluoroquinolone | Target Protection | 0.00E+00 | 0.00E+00 | 1.28E-04 | 0.00E+00 | 0.00E+00 | 0.00E+00 | 0.00E+00 | 0.00E+00 |
| 3003052 | *smeB* | Multidrug | Efflux | 1.64E-04 | 0.00E+00 | 7.99E-05 | 0.00E+00 | 0.00E+00 | 0.00E+00 | 0.00E+00 | 0.00E+00 |
| 3002639 | *aph(3'')-Ib* | Aminoglycoside | Inactivation | 0.00E+00 | 0.00E+00 | 0.00E+00 | 0.00E+00 | 0.00E+00 | 2.76E-04 | 0.00E+00 | 2.71E-04 |
| 3000656 | *acrS* | Multidrug | Efflux | 0.00E+00 | 0.00E+00 | 0.00E+00 | 0.00E+00 | 3.62E-04 | 0.00E+00 | 0.00E+00 | 0.00E+00 |
| 3000412 | *sul2* | Sulfonamide, sulfone | Target Replacement | 0.00E+00 | 1.25E-04 | 0.00E+00 | 0.00E+00 | 0.00E+00 | 4.08E-04 | 5.37E-04 | 0.00E+00 |
| 3002608 | *aadA8* | Aminoglycoside | Inactivation | 0.00E+00 | 0.00E+00 | 0.00E+00 | 0.00E+00 | 0.00E+00 | 1.12E-03 | 2.76E-04 | 0.00E+00 |
| 3000186 | *tetM* | Tetracycline | Target Protection | 0.00E+00 | 0.00E+00 | 0.00E+00 | 0.00E+00 | 0.00E+00 | 0.00E+00 | 0.00E+00 | 5.66E-05 |
| 3001328 | *mdfA* | Multidrug | Efflux | 0.00E+00 | 0.00E+00 | 0.00E+00 | 0.00E+00 | 9.73E-05 | 0.00E+00 | 0.00E+00 | 0.00E+00 |
| 3000237 | *tolC* | Multidrug | Efflux | 0.00E+00 | 0.00E+00 | 0.00E+00 | 0.00E+00 | 1.61E-04 | 0.00E+00 | 0.00E+00 | 0.00E+00 |
| 3003056 | *smeE* | Multidrug | Efflux | 4.97E-04 | 3.91E-04 | 4.30E-04 | 4.81E-04 | 1.54E-04 | 2.49E-04 | 3.51E-04 | 6.96E-05 |
| 3002711 | *qnrA5* | Fluoroquinolone | Target Protection | 0.00E+00 | 0.00E+00 | 1.28E-04 | 0.00E+00 | 0.00E+00 | 0.00E+00 | 0.00E+00 | 0.00E+00 |
| 3000522 | *ermG* | MLS | Target Alteration | 1.41E-04 | 0.00E+00 | 0.00E+00 | 0.00E+00 | 0.00E+00 | 9.05E-04 | 0.00E+00 | 0.00E+00 |
| 3000498 | *ermF* | MLS | Target Alteration | 0.00E+00 | 0.00E+00 | 0.00E+00 | 0.00E+00 | 7.49E-04 | 0.00E+00 | 0.00E+00 | 0.00E+00 |
| 3000190 | *tetO* | Tetracycline | Target Protection | 0.00E+00 | 0.00E+00 | 0.00E+00 | 0.00E+00 | 1.87E-04 | 5.78E-05 | 0.00E+00 | 2.27E-04 |
| 3000830 | *cpxA* | Multidrug | Efflux | 0.00E+00 | 0.00E+00 | 0.00E+00 | 0.00E+00 | 1.75E-04 | 0.00E+00 | 0.00E+00 | 0.00E+00 |
| 3000194 | *tetW* | Tetracycline | Target Protection | 0.00E+00 | 0.00E+00 | 0.00E+00 | 0.00E+00 | 6.25E-04 | 0.00E+00 | 1.14E-04 | 0.00E+00 |
| 3000491 | *acrD* | Aminoglycoside | Efflux | 2.66E-04 | 0.00E+00 | 1.35E-04 | 1.21E-04 | 3.08E-04 | 0.00E+00 | 0.00E+00 | 0.00E+00 |
| 3000593 | *ermQ* | MLS | Target Alteration | 0.00E+00 | 0.00E+00 | 0.00E+00 | 0.00E+00 | 3.10E-04 | 0.00E+00 | 0.00E+00 | 1.41E-04 |
| 3002879 | *linG* | Lincosamide | Inactivation | 0.00E+00 | 0.00E+00 | 0.00E+00 | 0.00E+00 | 0.00E+00 | 0.00E+00 | 2.66E-04 | 0.00E+00 |
| 3000502 | *acrF* | Multidrug | Efflux | 3.34E-05 | 0.00E+00 | 5.40E-05 | 3.02E-05 | 3.09E-04 | 3.57E-05 | 0.00E+00 | 0.00E+00 |
| 3002713 | *qnrA7* | Fluoroquinolone | Target Protection | 0.00E+00 | 0.00E+00 | 1.28E-04 | 0.00E+00 | 0.00E+00 | 0.00E+00 | 0.00E+00 | 0.00E+00 |
| 3000596 | *ermX* | MLS | Target Alteration | 0.00E+00 | 0.00E+00 | 0.00E+00 | 0.00E+00 | 0.00E+00 | 1.46E-04 | 0.00E+00 | 1.43E-04 |
| 3002836 | *lnuB* | Lincosamide | Inactivation | 7.73E-04 | 0.00E+00 | 0.00E+00 | 0.00E+00 | 0.00E+00 | 0.00E+00 | 1.09E-03 | 0.00E+00 |
| 3002637 | *aph(2'')-IVa* | Aminoglycoside | Inactivation | 0.00E+00 | 0.00E+00 | 0.00E+00 | 0.00E+00 | 0.00E+00 | 1.22E-04 | 0.00E+00 | 0.00E+00 |
| 3000775 | *adeB* | Tetracycline | Efflux | 0.00E+00 | 0.00E+00 | 2.70E-05 | 0.00E+00 | 0.00E+00 | 0.00E+00 | 0.00E+00 | 0.00E+00 |
| 3000567 | *tet(40)* | Tetracycline | Efflux | 0.00E+00 | 0.00E+00 | 0.00E+00 | 0.00E+00 | 1.96E-04 | 0.00E+00 | 3.59E-04 | 1.78E-04 |
| 3000795 | *mdtE* | Multidrug | Efflux | 0.00E+00 | 0.00E+00 | 0.00E+00 | 0.00E+00 | 4.14E-04 | 0.00E+00 | 1.89E-04 | 0.00E+00 |
| 3001396 | *oxa-1* | Beta-lactam | Inactivation | 0.00E+00 | 0.00E+00 | 1.01E-04 | 0.00E+00 | 0.00E+00 | 0.00E+00 | 0.00E+00 | 0.00E+00 |
| 3001405 | *oxa-10* | Beta-lactam | Inactivation | 0.00E+00 | 0.00E+00 | 0.00E+00 | 0.00E+00 | 0.00E+00 | 2.77E-04 | 0.00E+00 | 0.00E+00 |
| 3001710 | *oxa-211* | Beta-lactam | Inactivation | 0.00E+00 | 0.00E+00 | 0.00E+00 | 0.00E+00 | 0.00E+00 | 0.00E+00 | 0.00E+00 | 1.32E-04 |
| 3001813 | *oxa-55* | Beta-lactam | Inactivation | 0.00E+00 | 0.00E+00 | 2.31E-03 | 0.00E+00 | 0.00E+00 | 0.00E+00 | 0.00E+00 | 0.00E+00 |
| 3003175 | *carB-19* | Beta-lactam | Inactivation | 0.00E+00 | 0.00E+00 | 0.00E+00 | 0.00E+00 | 2.81E-04 | 0.00E+00 | 0.00E+00 | 0.00E+00 |
| 3001504 | *oxa-309* | Beta-lactam | Inactivation | 0.00E+00 | 0.00E+00 | 0.00E+00 | 0.00E+00 | 0.00E+00 | 0.00E+00 | 0.00E+00 | 1.32E-04 |
| 3002999 | *cblA-1* | Beta-lactam | Inactivation | 0.00E+00 | 0.00E+00 | 0.00E+00 | 0.00E+00 | 2.69E-04 | 0.00E+00 | 0.00E+00 | 0.00E+00 |
| 3001425 | *oxa-31* | Beta-lactam | Inactivation | 0.00E+00 | 0.00E+00 | 1.01E-04 | 0.00E+00 | 0.00E+00 | 0.00E+00 | 0.00E+00 | 0.00E+00 |
| 3003176 | *carB-21* | Beta-lactam | Inactivation | 0.00E+00 | 1.09E-04 | 0.00E+00 | 0.00E+00 | 0.00E+00 | 0.00E+00 | 0.00E+00 | 0.00E+00 |
| 3001798 | *oxa-74* | Beta-lactam | Inactivation | 0.00E+00 | 0.00E+00 | 0.00E+00 | 0.00E+00 | 0.00E+00 | 1.38E-04 | 0.00E+00 | 0.00E+00 |
| 3002875 | *dfrE* | Diaminopyrimidine | Target Replacement | 0.00E+00 | 2.05E-04 | 8.47E-04 | 5.69E-04 | 0.00E+00 | 2.24E-04 | 2.21E-04 | 2.20E-04 |
| 3002242 | *carB-3* | Beta-lactam | Inactivation | 0.00E+00 | 0.00E+00 | 0.00E+00 | 1.03E-04 | 0.00E+00 | 0.00E+00 | 0.00E+00 | 0.00E+00 |
| 3001417 | *oxa-22* | Beta-lactam | Inactivation | 0.00E+00 | 0.00E+00 | 4.07E-04 | 0.00E+00 | 0.00E+00 | 0.00E+00 | 2.65E-04 | 0.00E+00 |
| 3002250 | *carB-12* | Beta-lactam | Inactivation | 0.00E+00 | 2.34E-04 | 0.00E+00 | 0.00E+00 | 0.00E+00 | 0.00E+00 | 0.00E+00 | 0.00E+00 |
| 3003284 | *rpoB* | Multidrug | Target Alteration | 2.77E-02 | 2.63E-02 | 2.41E-02 | 2.26E-02 | 3.00E-02 | 3.08E-02 | 3.15E-02 | 3.21E-02 |
| 3003294 | *gyrA* | Fluoroquinolone | Target Alteration | 1.30E-02 | 8.24E-03 | 1.11E-02 | 9.61E-03 | 1.20E-02 | 1.46E-02 | 1.23E-02 | 1.37E-02 |
| 3003291 | *rpoC* | Peptide | Target Alteration | 2.00E-03 | 2.86E-03 | 2.29E-03 | 3.06E-03 | 2.35E-03 | 3.83E-03 | 3.81E-03 | 3.93E-03 |
| 3003301 | *gyrB* | Aminocoumarin | Target Alteration | 1.42E-02 | 9.00E-03 | 1.02E-02 | 1.14E-02 | 1.34E-02 | 1.29E-02 | 1.08E-02 | 1.29E-02 |
| 3003316 | *parE* | Fluoroquinolone | Target Alteration | 6.62E-03 | 1.40E-03 | 2.44E-03 | 2.98E-03 | 2.47E-03 | 3.93E-03 | 2.54E-03 | 2.93E-03 |
| 3003327 | *embC* | Polyamine | Target Alteration | 0.00E+00 | 1.24E-04 | 0.00E+00 | 0.00E+00 | 0.00E+00 | 0.00E+00 | 6.67E-05 | 0.00E+00 |
| 3003453 | *embA* | Polyamine | Target Alteration | 0.00E+00 | 0.00E+00 | 0.00E+00 | 5.72E-05 | 0.00E+00 | 0.00E+00 | 0.00E+00 | 0.00E+00 |
| 3003463 | *kasA* | Unclassified antibiotic | Target Alteration | 2.48E-04 | 0.00E+00 | 0.00E+00 | 0.00E+00 | 9.59E-05 | 1.77E-04 | 8.75E-05 | 0.00E+00 |
| 3003393 | *inhA* | Unclassified antibiotic | Target Alteration | 0.00E+00 | 0.00E+00 | 0.00E+00 | 0.00E+00 | 0.00E+00 | 0.00E+00 | 1.35E-04 | 0.00E+00 |
| 3003461 | *ndh* | Unclassified antibiotic | Target Alteration | 0.00E+00 | 0.00E+00 | 1.20E-04 | 0.00E+00 | 8.61E-05 | 5.58E-04 | 7.87E-05 | 2.34E-04 |
| 3003395 | *rpsL* | Multidrug | Target Alteration | 4.42E-03 | 7.05E-03 | 3.35E-03 | 3.00E-03 | 1.06E-02 | 1.18E-02 | 6.72E-03 | 1.02E-02 |
| 3003308 | *parC* | Fluoroquinolone | Target Alteration | 9.17E-03 | 4.86E-03 | 6.31E-03 | 5.69E-03 | 9.02E-03 | 8.49E-03 | 8.97E-03 | 9.77E-03 |
| 3003577 | *pmrE* | Peptide | Target Alteration | 2.66E-04 | 1.74E-04 | 1.08E-03 | 2.41E-04 | 6.17E-04 | 1.90E-04 | 1.88E-04 | 4.66E-04 |
| 3003576 | *pmrC* | Peptide | Target Alteration | 0.00E+00 | 0.00E+00 | 0.00E+00 | 0.00E+00 | 7.29E-05 | 0.00E+00 | 0.00E+00 | 0.00E+00 |
| 3003610 | *oxa-454* | Beta-lactam | Inactivation | 0.00E+00 | 0.00E+00 | 0.00E+00 | 0.00E+00 | 0.00E+00 | 5.54E-04 | 0.00E+00 | 0.00E+00 |
| 3003206 | *lsaE* | Multidrug | Efflux | 0.00E+00 | 0.00E+00 | 0.00E+00 | 0.00E+00 | 0.00E+00 | 0.00E+00 | 1.47E-04 | 0.00E+00 |
| 3003550 | *mdtP* | Multidrug | Efflux | 0.00E+00 | 0.00E+00 | 0.00E+00 | 0.00E+00 | 1.63E-04 | 0.00E+00 | 0.00E+00 | 0.00E+00 |
| 3003465 | *embB* | Polyamine | Target Alteration | 0.00E+00 | 0.00E+00 | 0.00E+00 | 2.85E-05 | 0.00E+00 | 6.73E-05 | 0.00E+00 | 0.00E+00 |
| 3002841 | *vatB* | Streptogramin | Inactivation | 0.00E+00 | 0.00E+00 | 0.00E+00 | 0.00E+00 | 3.75E-04 | 0.00E+00 | 0.00E+00 | 0.00E+00 |
| 3002870 | *tet34* | Tetracycline | Unknown | 3.38E-04 | 1.11E-04 | 0.00E+00 | 5.11E-04 | 0.00E+00 | 0.00E+00 | 0.00E+00 | 0.00E+00 |
| 3002712 | *qnrA6* | Fluoroquinolone | Target Protection | 0.00E+00 | 0.00E+00 | 2.55E-04 | 0.00E+00 | 0.00E+00 | 0.00E+00 | 0.00E+00 | 0.00E+00 |
| 3003369 | *ef-Tu* | Elfamycin | Target Alteration | 8.84E-02 | 7.87E-02 | 7.45E-02 | 8.11E-02 | 9.40E-02 | 1.02E-01 | 9.03E-02 | 9.48E-02 |
| 3002613 | *aadA13* | Aminoglycoside | Inactivation | 0.00E+00 | 0.00E+00 | 0.00E+00 | 0.00E+00 | 0.00E+00 | 0.00E+00 | 0.00E+00 | 2.73E-04 |
| 3003682 | *opmH* | Unclassified antibiotic | Efflux | 0.00E+00 | 0.00E+00 | 5.79E-05 | 0.00E+00 | 0.00E+00 | 7.65E-05 | 0.00E+00 | 0.00E+00 |
| 3002626 | *ant(6)-Ia* | Aminoglycoside | Inactivation | 0.00E+00 | 0.00E+00 | 9.71E-05 | 0.00E+00 | 0.00E+00 | 0.00E+00 | 0.00E+00 | 0.00E+00 |
| 3003728 | *vanRI* | Glycopeptide | Target Alteration | 0.00E+00 | 0.00E+00 | 0.00E+00 | 0.00E+00 | 0.00E+00 | 0.00E+00 | 3.13E-04 | 0.00E+00 |
| 3003031 | *mexW* | Multidrug | Efflux | 0.00E+00 | 0.00E+00 | 2.74E-04 | 3.07E-05 | 0.00E+00 | 1.09E-04 | 0.00E+00 | 0.00E+00 |
| 3003693 | *mexK* | Multidrug | Efflux | 3.36E-05 | 0.00E+00 | 2.72E-04 | 3.05E-05 | 0.00E+00 | 3.60E-05 | 3.56E-05 | 0.00E+00 |
| 3003392 | *katG* | Unclassified antibiotic | Target Alteration | 4.19E-04 | 3.66E-04 | 2.26E-04 | 4.22E-04 | 3.78E-04 | 4.99E-04 | 3.45E-04 | 4.89E-04 |
| 3003742 | *mphG* | Macrolide | Inactivation | 0.00E+00 | 1.15E-04 | 0.00E+00 | 0.00E+00 | 0.00E+00 | 0.00E+00 | 0.00E+00 | 0.00E+00 |
| 3003748 | *oleC* | Macrolide | Efflux | 0.00E+00 | 0.00E+00 | 0.00E+00 | 0.00E+00 | 0.00E+00 | 0.00E+00 | 1.12E-04 | 0.00E+00 |
| 3003751 | *nfsA* | Nitrofuran | Target Alteration | 0.00E+00 | 0.00E+00 | 1.16E-04 | 0.00E+00 | 1.66E-04 | 0.00E+00 | 0.00E+00 | 0.00E+00 |
| 3003730 | *ileS* | Unclassified antibiotic | Target Alteration | 0.00E+00 | 0.00E+00 | 0.00E+00 | 0.00E+00 | 0.00E+00 | 0.00E+00 | 3.29E-05 | 0.00E+00 |
| 3003784 | *murA* | Unclassified antibiotic | Target Alteration | 5.75E-04 | 3.23E-04 | 6.66E-05 | 1.49E-04 | 3.81E-04 | 6.16E-04 | 7.82E-04 | 0.00E+00 |
| 3003318 | *parY* | Aminocoumarin | Target Alteration | 4.90E-05 | 1.93E-04 | 1.59E-04 | 1.78E-04 | 2.27E-04 | 3.15E-04 | 3.11E-04 | 2.06E-04 |
| 3003382 | *soxR* | Multidrug | Efflux | 0.00E+00 | 0.00E+00 | 0.00E+00 | 0.00E+00 | 5.23E-04 | 0.00E+00 | 0.00E+00 | 0.00E+00 |
| 3003835 | *cdeA* | Multidrug | Efflux | 0.00E+00 | 0.00E+00 | 0.00E+00 | 0.00E+00 | 1.81E-04 | 0.00E+00 | 0.00E+00 | 0.00E+00 |
| 3003838 | *gadW* | Multidrug | Efflux | 0.00E+00 | 0.00E+00 | 0.00E+00 | 0.00E+00 | 6.58E-04 | 0.00E+00 | 0.00E+00 | 0.00E+00 |
| 3003843 | *leuO* | Multidrug | Efflux | 0.00E+00 | 1.08E-04 | 3.55E-04 | 0.00E+00 | 0.00E+00 | 0.00E+00 | 1.16E-04 | 0.00E+00 |
| 3003844 | *mfd* | Fluoroquinolone | Target Protection | 2.40E-04 | 2.65E-04 | 4.38E-04 | 1.63E-04 | 4.17E-04 | 1.61E-04 | 3.49E-04 | 2.84E-04 |
| 3000309 | *emrD* | Multidrug | Efflux | 0.00E+00 | 0.00E+00 | 0.00E+00 | 0.00E+00 | 4.05E-04 | 0.00E+00 | 0.00E+00 | 0.00E+00 |
| 3003889 | *glpT* | Unclassified antibiotic | Target Alteration | 0.00E+00 | 0.00E+00 | 0.00E+00 | 0.00E+00 | 3.53E-04 | 0.00E+00 | 0.00E+00 | 0.00E+00 |
| 3003890 | *uhpT* | Unclassified antibiotic | Target Alteration | 0.00E+00 | 0.00E+00 | 1.20E-04 | 0.00E+00 | 2.58E-04 | 0.00E+00 | 0.00E+00 | 0.00E+00 |
| 3003895 | *phoP* | Multidrug | Target Alteration | 0.00E+00 | 0.00E+00 | 1.24E-04 | 0.00E+00 | 0.00E+00 | 0.00E+00 | 0.00E+00 | 0.00E+00 |
| 3002578 | *aac(6')-Ib7* | Aminoglycoside | Inactivation | 0.00E+00 | 0.00E+00 | 8.56E-05 | 0.00E+00 | 2.45E-04 | 2.26E-04 | 1.12E-04 | 0.00E+00 |
| 3002831 | *vgaC* | Multidrug | Efflux | 0.00E+00 | 0.00E+00 | 0.00E+00 | 0.00E+00 | 5.19E-04 | 0.00E+00 | 0.00E+00 | 0.00E+00 |
| 3003150 | *carB-20* | Beta-lactam | Inactivation | 0.00E+00 | 9.66E-05 | 0.00E+00 | 0.00E+00 | 0.00E+00 | 0.00E+00 | 0.00E+00 | 0.00E+00 |
| 3002676 | *catB3* | Phenicol | Inactivation | 0.00E+00 | 1.61E-04 | 1.32E-04 | 2.97E-04 | 0.00E+00 | 0.00E+00 | 0.00E+00 | 0.00E+00 |
| 3001555 | *oxa-368* | Beta-lactam | Inactivation | 0.00E+00 | 0.00E+00 | 0.00E+00 | 0.00E+00 | 0.00E+00 | 4.08E-05 | 0.00E+00 | 0.00E+00 |
| 3003923 | *oqxB* | Multidrug | Efflux | 0.00E+00 | 3.22E-05 | 0.00E+00 | 0.00E+00 | 0.00E+00 | 0.00E+00 | 0.00E+00 | 0.00E+00 |
| 3003950 | *msbA* | Nitroimidazole | Efflux | 1.18E-04 | 2.32E-04 | 0.00E+00 | 1.07E-04 | 0.00E+00 | 0.00E+00 | 0.00E+00 | 6.22E-05 |
| 3003955 | *efpA* | Multidrug | Efflux | 2.60E-04 | 6.38E-05 | 0.00E+00 | 0.00E+00 | 0.00E+00 | 2.09E-04 | 1.37E-04 | 6.83E-05 |
| 3003952 | *yojI* | Peptide | Efflux | 0.00E+00 | 0.00E+00 | 0.00E+00 | 0.00E+00 | 2.19E-04 | 0.00E+00 | 0.00E+00 | 0.00E+00 |
| 3003980 | *tetA(48)* | Tetracycline | Efflux | 0.00E+00 | 0.00E+00 | 0.00E+00 | 0.00E+00 | 0.00E+00 | 0.00E+00 | 1.06E-04 | 0.00E+00 |
| 3003992 | *rphB* | Rifamycin | Inactivation | 3.90E-05 | 0.00E+00 | 0.00E+00 | 0.00E+00 | 0.00E+00 | 1.25E-04 | 0.00E+00 | 0.00E+00 |
| 3003741 | *mphD* | Macrolide | Inactivation | 0.00E+00 | 0.00E+00 | 0.00E+00 | 0.00E+00 | 0.00E+00 | 0.00E+00 | 1.24E-04 | 0.00E+00 |
| 3004043 | *acrA* | Multidrug | Efflux | 0.00E+00 | 0.00E+00 | 0.00E+00 | 0.00E+00 | 2.01E-04 | 0.00E+00 | 0.00E+00 | 0.00E+00 |
| 3004049 | *fabG* | Unclassified antibiotic | Target Alteration | 1.41E-04 | 4.15E-04 | 0.00E+00 | 0.00E+00 | 3.26E-04 | 0.00E+00 | 0.00E+00 | 2.96E-04 |
| 3000216 | *acrB* | Multidrug | Efflux | 3.62E-04 | 6.45E-05 | 5.32E-05 | 3.58E-04 | 3.81E-05 | 7.04E-05 | 1.74E-04 | 3.45E-05 |
| 3004054 | *cpxR* | Multidrug | Efflux | 6.11E-04 | 0.00E+00 | 4.95E-04 | 0.00E+00 | 0.00E+00 | 3.27E-04 | 0.00E+00 | 0.00E+00 |
| 3004122 | *ompK37* | Beta-lactam | Reduced Permeability | 1.84E-04 | 0.00E+00 | 0.00E+00 | 0.00E+00 | 0.00E+00 | 0.00E+00 | 0.00E+00 | 0.00E+00 |
| 3004126 | *lamB* | Multidrug | Reduced Permeability | 0.00E+00 | 0.00E+00 | 0.00E+00 | 0.00E+00 | 1.79E-04 | 0.00E+00 | 0.00E+00 | 0.00E+00 |

**Supplementary Table S2.** ARG abundance according to resistance type.

| Resistance type | Mangrove area | | | |  | Non-mangrove area | | |
| --- | --- | --- | --- | --- | --- | --- | --- | --- |
|  | A | B | C | D | E | X | Y | Z |
| Elfamycin | 8.84E-02 | 7.87E-02 | 7.45E-02 | 8.11E-02 | 9.40E-02 | 1.02E-01 | 9.03E-02 | 9.48E-02 |
| Multidrug | 3.76E-02 | 3.45E-02 | 3.38E-02 | 2.79E-02 | 4.75E-02 | 4.52E-02 | 4.19E-02 | 4.40E-02 |
| Fluoroquinolone | 2.91E-02 | 1.51E-02 | 2.13E-02 | 1.84E-02 | 2.45E-02 | 2.71E-02 | 2.42E-02 | 2.67E-02 |
| Aminocoumarin | 1.43E-02 | 9.19E-03 | 1.04E-02 | 1.16E-02 | 1.39E-02 | 1.32E-02 | 1.12E-02 | 1.32E-02 |
| Peptide | 2.74E-03 | 3.39E-03 | 3.54E-03 | 3.86E-03 | 3.56E-03 | 4.13E-03 | 3.99E-03 | 4.51E-03 |
| Unclassified antibiotic | 1.38E-03 | 1.10E-03 | 5.92E-04 | 5.71E-04 | 1.88E-03 | 1.93E-03 | 1.46E-03 | 1.02E-03 |
| Tetracycline | 9.46E-04 | 4.89E-04 | 7.82E-04 | 6.80E-04 | 2.12E-03 | 1.93E-03 | 9.93E-04 | 1.10E-03 |
| Aminoglycoside | 7.03E-04 | 0.00E+00 | 3.17E-04 | 1.21E-04 | 1.04E-03 | 3.04E-03 | 1.47E-03 | 1.09E-03 |
| Beta-lactam | 1.84E-04 | 4.40E-04 | 2.92E-03 | 1.03E-04 | 5.51E-04 | 1.01E-03 | 2.65E-04 | 2.64E-04 |
| Sulfonamide, sulfone | 1.23E-04 | 2.46E-04 | 0.00E+00 | 0.00E+00 | 0.00E+00 | 9.36E-04 | 2.23E-03 | 1.29E-04 |
| Lincosamide | 8.99E-04 | 0.00E+00 | 0.00E+00 | 0.00E+00 | 0.00E+00 | 8.07E-04 | 1.58E-03 | 0.00E+00 |
| MLS | 1.41E-04 | 0.00E+00 | 0.00E+00 | 0.00E+00 | 1.22E-03 | 1.05E-03 | 0.00E+00 | 2.83E-04 |
| Diaminopyrimidine | 0.00E+00 | 2.05E-04 | 8.47E-04 | 5.69E-04 | 0.00E+00 | 2.24E-04 | 2.21E-04 | 2.20E-04 |
| Phenicol | 1.64E-04 | 3.22E-04 | 1.32E-04 | 4.46E-04 | 2.13E-04 | 0.00E+00 | 3.72E-04 | 7.62E-05 |
| Macrolide | 0.00E+00 | 1.15E-04 | 0.00E+00 | 6.41E-05 | 0.00E+00 | 5.45E-04 | 4.14E-04 | 0.00E+00 |
| Nitroimidazole | 1.18E-04 | 2.32E-04 | 0.00E+00 | 1.07E-04 | 0.00E+00 | 0.00E+00 | 0.00E+00 | 6.22E-05 |
| Rifamycin | 3.90E-05 | 0.00E+00 | 2.43E-04 | 0.00E+00 | 0.00E+00 | 1.25E-04 | 0.00E+00 | 0.00E+00 |
| Streptogramin | 0.00E+00 | 0.00E+00 | 0.00E+00 | 0.00E+00 | 3.75E-04 | 0.00E+00 | 0.00E+00 | 0.00E+00 |
| Polyamine | 0.00E+00 | 0.00E+00 | 1.24E-04 | 0.00E+00 | 8.56E-05 | 0.00E+00 | 6.73E-05 | 6.67E-05 |
| Glycopeptide | 0.00E+00 | 0.00E+00 | 0.00E+00 | 0.00E+00 | 0.00E+00 | 0.00E+00 | 3.13E-04 | 0.00E+00 |
| Nitrofuran | 0.00E+00 | 0.00E+00 | 1.16E-04 | 0.00E+00 | 1.66E-04 | 0.00E+00 | 0.00E+00 | 0.00E+00 |
| Nucleoside | 0.00E+00 | 0.00E+00 | 0.00E+00 | 0.00E+00 | 2.21E-04 | 0.00E+00 | 0.00E+00 | 0.00E+00 |

**Supplementary Table S3.** ARG abundance according to resistance mechanism.

| Resistance mechanism | Mangrove area | | | |  | Non-mangrove area | | |
| --- | --- | --- | --- | --- | --- | --- | --- | --- |
|  | A | B | C | D | E | X | Y | Z |
| Target Alteration | 1.68E-01 | 1.40E-01 | 1.36E-01 | 1.41E-01 | 1.78E-01 | 1.92E-01 | 1.69E-01 | 1.82E-01 |
| Efflux | 6.53E-03 | 1.68E-03 | 7.29E-03 | 2.69E-03 | 9.19E-03 | 3.74E-03 | 5.12E-03 | 2.39E-03 |
| Inactivation | 1.54E-03 | 8.77E-04 | 3.24E-03 | 5.48E-04 | 1.88E-03 | 5.52E-03 | 3.62E-03 | 1.45E-03 |
| Target Protection | 3.64E-04 | 1.13E-03 | 2.55E-03 | 7.32E-04 | 1.60E-03 | 2.18E-03 | 2.97E-03 | 1.19E-03 |
| Target Replacement | 1.23E-04 | 4.51E-04 | 8.47E-04 | 5.69E-04 | 0.00E+00 | 1.16E-03 | 2.45E-03 | 3.49E-04 |
| Unknown | 3.38E-04 | 1.11E-04 | 0.00E+00 | 5.11E-04 | 0.00E+00 | 0.00E+00 | 0.00E+00 | 0.00E+00 |
| Reduced Permeability | 1.84E-04 | 0.00E+00 | 0.00E+00 | 0.00E+00 | 1.79E-04 | 0.00E+00 | 0.00E+00 | 0.00E+00 |

**Supplementary Table S4.** The values of sediment properties.

| Sediment properties | Mangrove area | | | |  | Non-mangrove area | | |
| --- | --- | --- | --- | --- | --- | --- | --- | --- |
|  | A | B | C | D | E | X | Y | Z |
| pH | 6.5 | 6.3 | 8.1 | 5.7 | 8.2 | 7.5 | 8.4 | 8.2 |
| TOC (g/kg) | 16.0 | 24.8 | 16.1 | 22.9 | 10.4 | 7.05 | 6.84 | 7.01 |
| Cu (mg/kg) | 15.1 | 16.5 | 12.9 | 25.9 | 12.6 | 30.4 | 12.8 | 12.7 |
| Zn (mg/kg) | 39.5 | 43.9 | 43.3 | 38.2 | 39.0 | 52.9 | 32.7 | 47.3 |
| As (mg/kg) | 9.17 | 6.84 | 7.42 | 8.54 | 5.58 | 2.08 | 1.89 | 1.9 |
| Hg (mg/kg) | 0.0407 | 0.0443 | 0.0347 | 0.0377 | 0.0361 | 0.0252 | 0.0164 | 0.0273 |
| Cd (mg/kg) | 0.0558 | 0.0712 | 0.067 | 0.0558 | 0.0525 | 0.0352 | 0.037 | 0.0275 |
